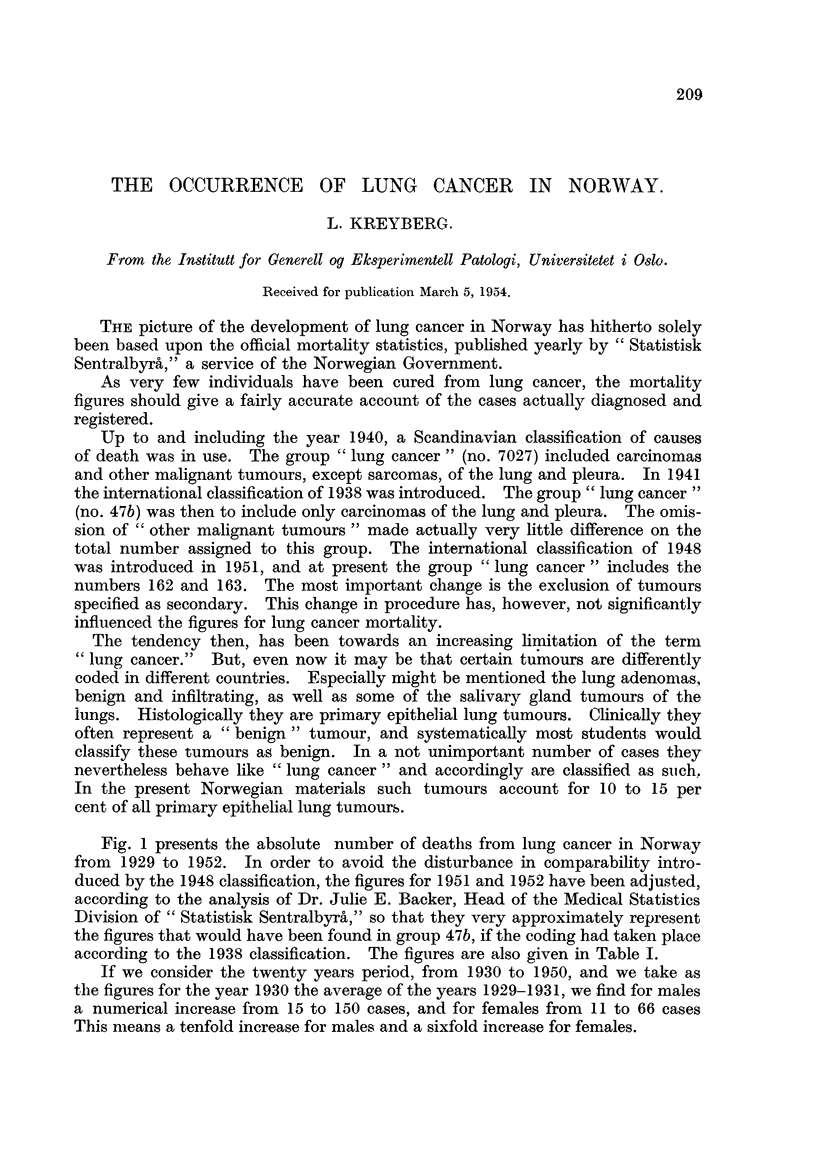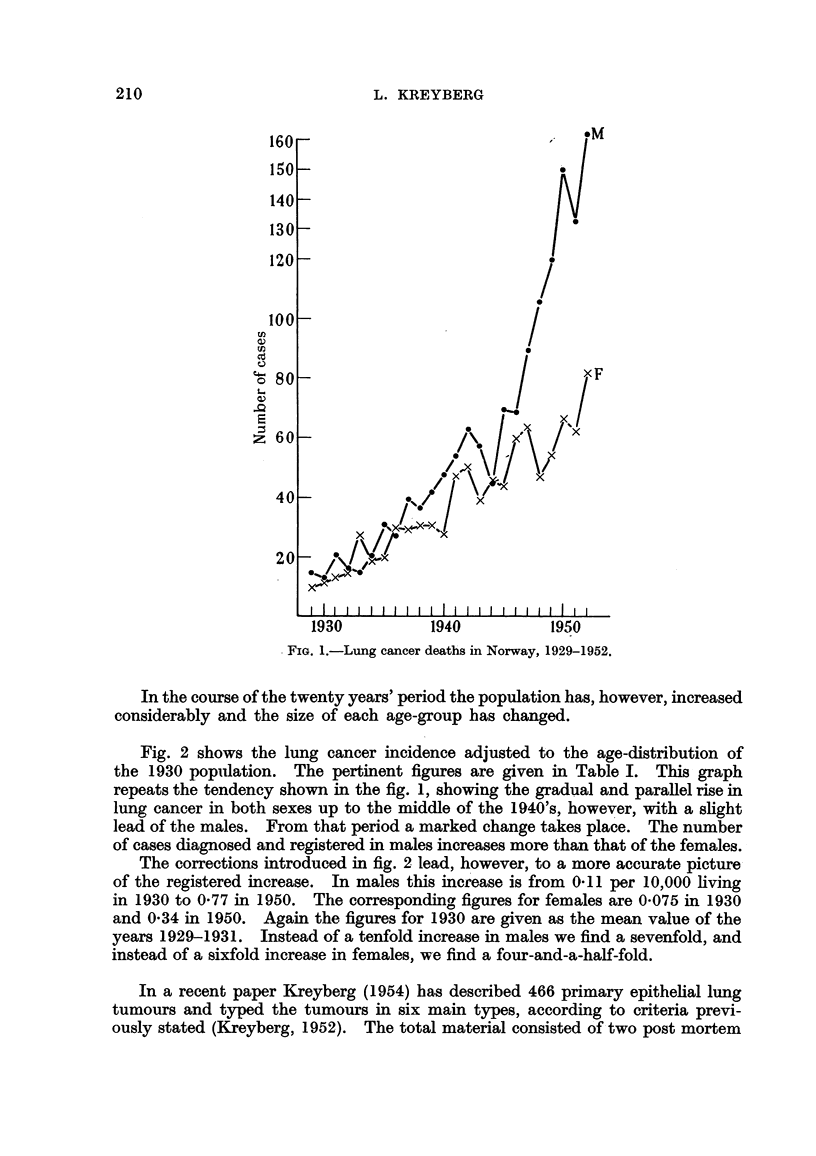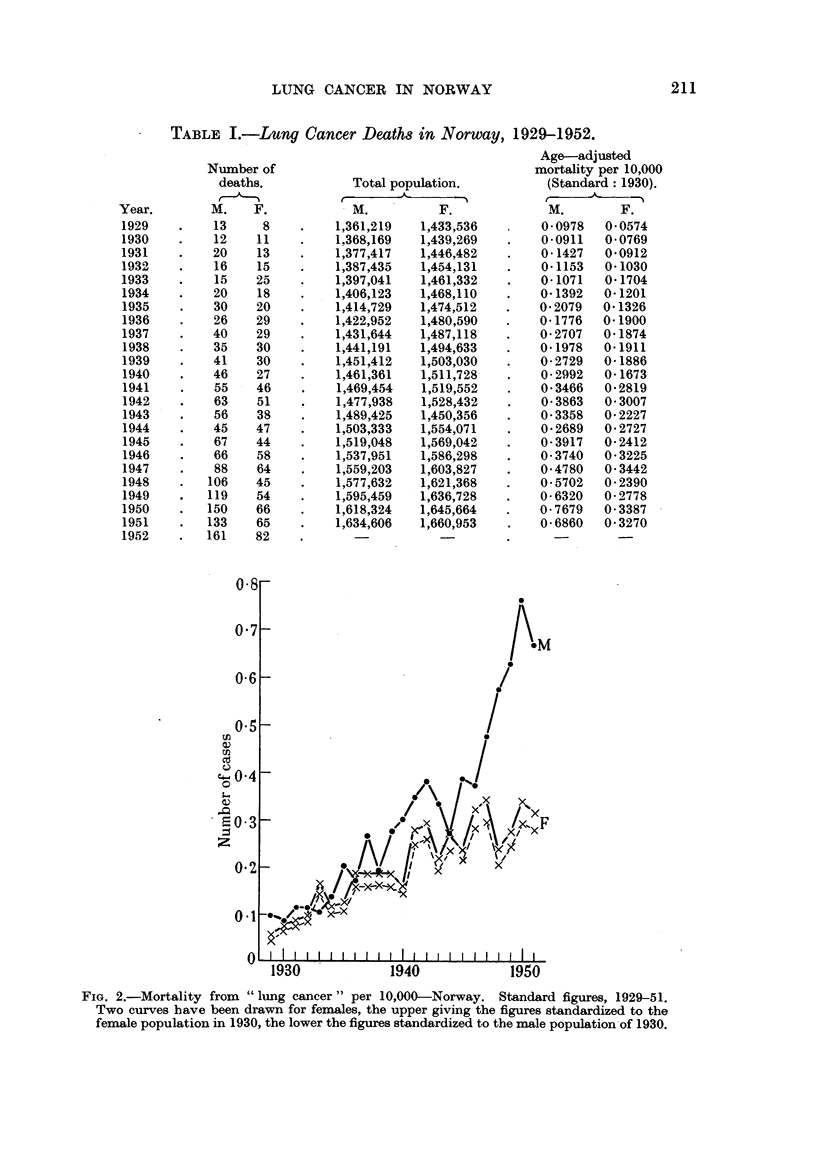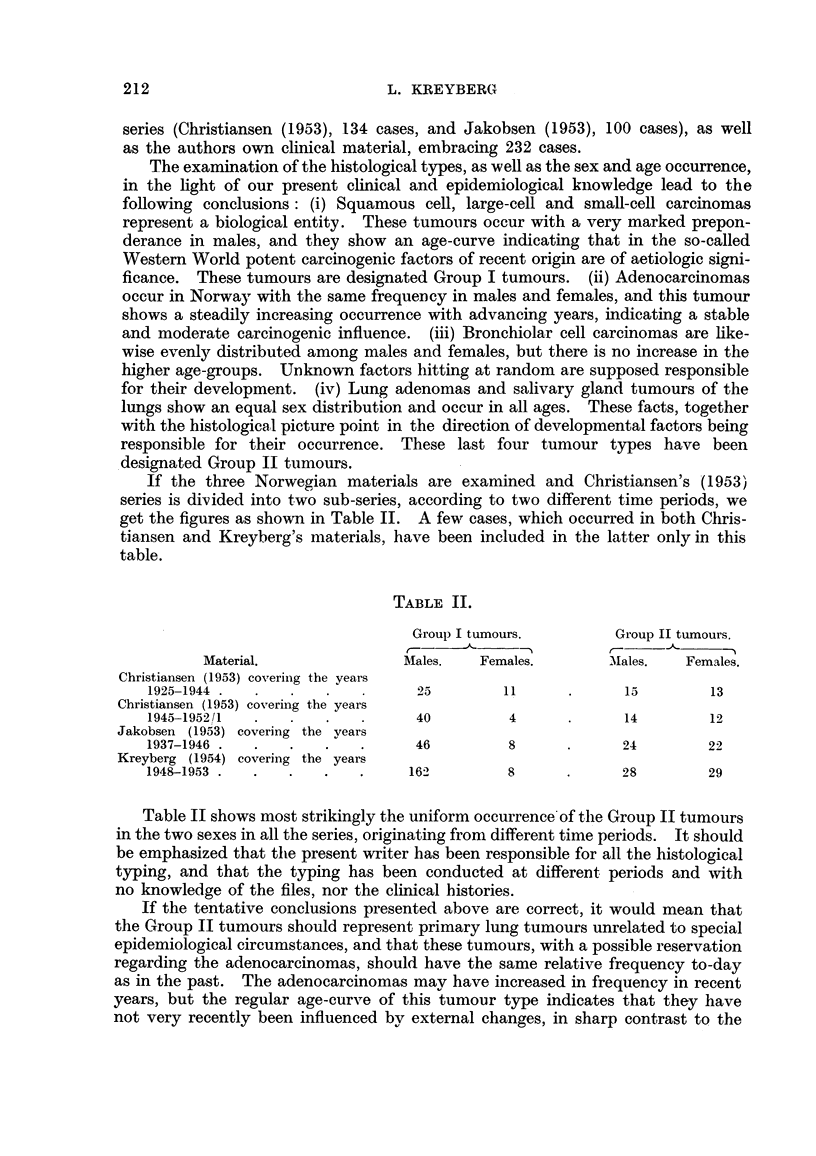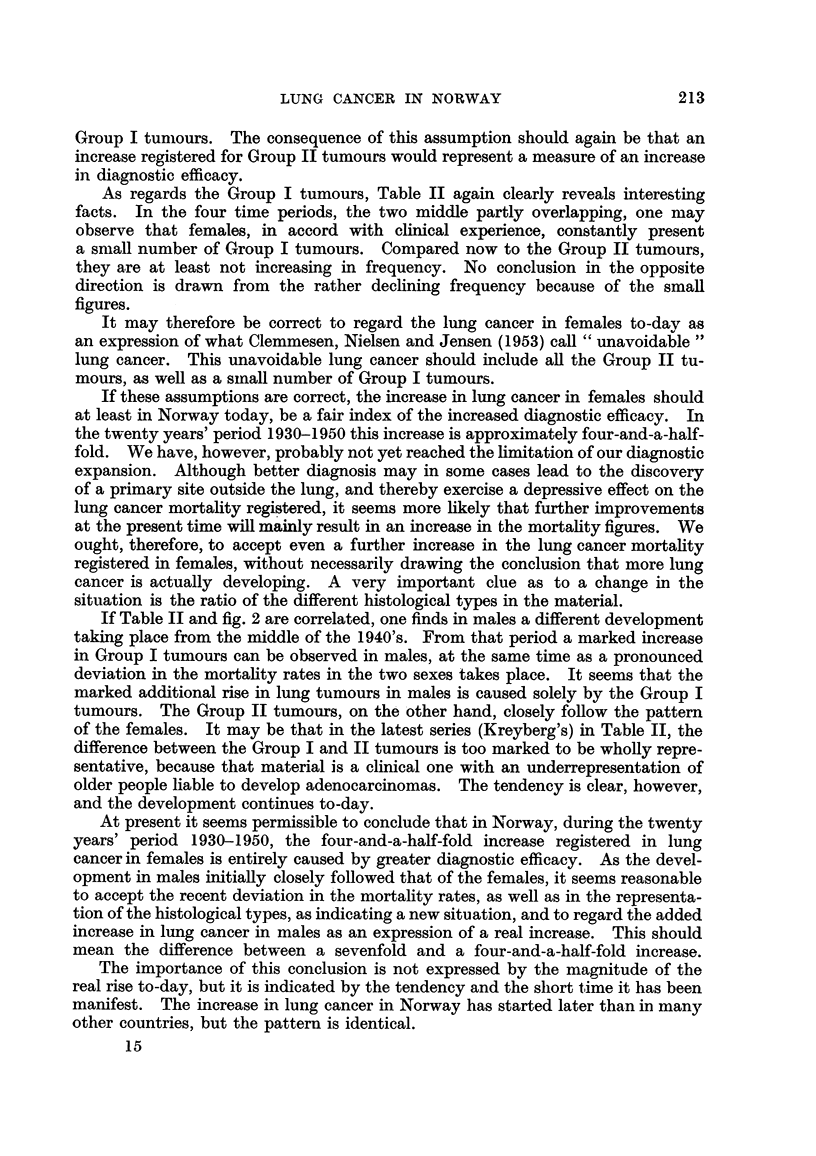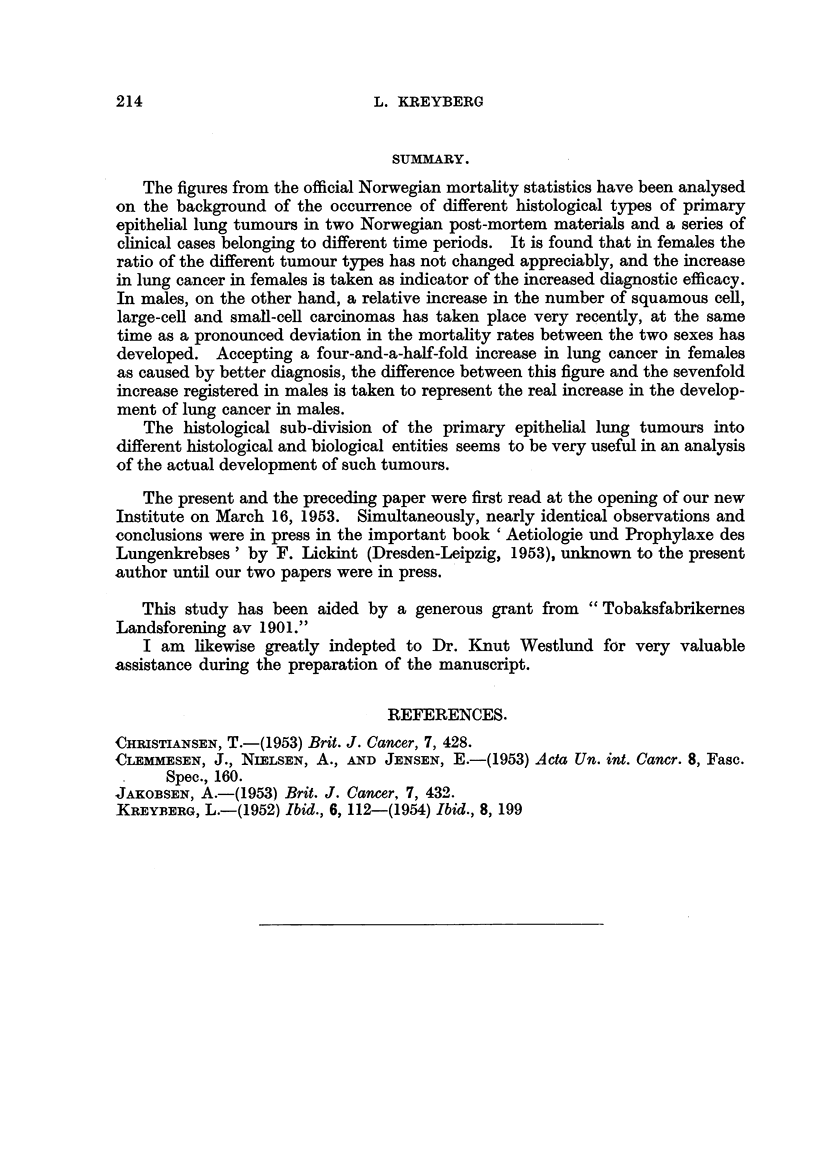# The Occurrence of Lung Cancer in Norway

**DOI:** 10.1038/bjc.1954.20

**Published:** 1954-06

**Authors:** L. Kreyberg


### THE OCCURRENCE OF LUNG CANCER IN NORWAY.

L. KREYBERG.

*From the Institutt for Generell og Eksperimentell Patologi, Universitetet i Oslo.*

Received for publication March 5, 1954.

THE picture of the development of lung cancer in Norway has hitherto solely been based upon the official mortality statistics, published yearly by " Statistisk Sentralbyrå," a service of the Norwegian Government.

As very few individuals have been cured from lung cancer, the mortality figures should give a fairly accurate account of the cases actually diagnosed and registered.

Up to and including the year 1940, a Scandinavian classification of causes of death was in use. The group " lung cancer " (no. 7027) included carcinomas and other malignant tumours, except sarcomas, of the lung and pleura. In 1941 the international classification of 1938 was introduced. The group " lung cancer " (no. 47*b*) was then to include only carcinomas of the lung and pleura. The omission of " other malignant tumours " made actually very little difference on the total number assigned to this group. The international classification of 1948 was introduced in 1951, and at present the group " lung cancer " includes the numbers 162 and 163. The most important change is the exclusion of tumours specified as secondary. This change in procedure has, however, not significantly influenced the figures for lung cancer mortality.

The tendency then, has been towards an increasing limitation of the term " lung cancer." But, even now it may be that certain tumours are differently coded in different countries. Especially might be mentioned the lung adenomas, benign and infiltrating, as well as some of the salivary gland tumours of the lungs. Histologically they are primary epithelial lung tumours. Clinically they often represent a " benign " tumour, and systematically most students would classify these tumours as benign. In a not unimportant number of cases they nevertheless behave like " lung cancer " and accordingly are classified as such. In the present Norwegian materials such tumours account for 10 to 15 per cent of all primary epithelial lung tumours.

Fig. 1 presents the absolute number of deaths from lung cancer in Norway from 1929 to 1952. In order to avoid the disturbance in comparability introduced by the 1948 classification, the figures for 1951 and 1952 have been adjusted, according to the analysis of Dr. Julie E. Backer, Head of the Medical Statistics Division of " Statistisk Sentralbyrå," so that they very approximately represent the figures that would have been found in group 47*b*, if the coding had taken place according to the 1938 classification. The figures are also given in Table I.

If we consider the twenty years period, from 1930 to 1950, and we take as the figures for the year 1930 the average of the years 1929–1931, we find for males a numerical increase from 15 to 150 cases, and for females from 11 to 66 cases This means a tenfold increase for males and a sixfold increase for females.

FIG. 1.—Lung cancer deaths in Norway, 1929–1952.

In the course of the twenty years' period the population has, however, increased considerably and the size of each age-group has changed.

Fig. 2 shows the lung cancer incidence adjusted to the age-distribution of the 1930 population. The pertinent figures are given in Table I. This graph repeats the tendency shown in the fig. 1, showing the gradual and parallel rise in lung cancer in both sexes up to the middle of the 1940's, however, with a slight lead of the males. From that period a marked change takes place. The number of cases diagnosed and registered in males increases more than that of the females.

The corrections introduced in fig. 2 lead, however, to a more accurate picture of the registered increase. In males this increase is from 0·11 per 10,000 living in 1930 to 0·77 in 1950. The corresponding figures for females are 0·075 in 1930 and 0·34 in 1950. Again the figures for 1930 are given as the mean value of the years 1929–1931. Instead of a tenfold increase in males we find a sevenfold, and instead of a sixfold increase in females, we find a four-and-a-half-fold.

In a recent paper Kreyberg (1954) has described 466 primary epithelial lung tumours and typed the tumours in six main types, according to criteria previously stated (Kreyberg, 1952). The total material consisted of two post mortem

TABLE I.—*Lung Cancer Deaths in Norway*, 1929–1952.

| Year. | Number of deaths. M. | Number of deaths. F. | Total population. M. | Total population. F. | Age—adjusted mortality per 10,000 (Standard : 1930). M. | Age—adjusted mortality per 10,000 (Standard : 1930). F. |
|---|---|---|---|---|---|---|
| 1929 | 13 | 8 | 1,361,219 | 1,433,536 | 0·0978 | 0·0574 |
| 1930 | 12 | 11 | 1,368,169 | 1,439,269 | 0·0911 | 0·0769 |
| 1931 | 20 | 13 | 1,377,417 | 1,446,482 | 0·1427 | 0·0912 |
| 1932 | 16 | 15 | 1,387,435 | 1,454,131 | 0·1153 | 0·1030 |
| 1933 | 15 | 25 | 1,397,041 | 1,461,332 | 0·1071 | 0·1704 |
| 1934 | 20 | 18 | 1,406,123 | 1,468,110 | 0·1392 | 0·1201 |
| 1935 | 30 | 20 | 1,414,729 | 1,474,512 | 0·2079 | 0·1326 |
| 1936 | 26 | 29 | 1,422,952 | 1,480,590 | 0·1776 | 0·1900 |
| 1937 | 40 | 29 | 1,431,644 | 1,487,118 | 0·2707 | 0·1874 |
| 1938 | 35 | 30 | 1,441,191 | 1,494,633 | 0·1978 | 0·1911 |
| 1939 | 41 | 30 | 1,451,412 | 1,503,030 | 0·2729 | 0·1886 |
| 1940 | 46 | 27 | 1,461,361 | 1,511,728 | 0·2992 | 0·1673 |
| 1941 | 55 | 46 | 1,469,454 | 1,519,552 | 0·3466 | 0·2819 |
| 1942 | 63 | 51 | 1,477,938 | 1,528,432 | 0·3863 | 0·3007 |
| 1943 | 56 | 38 | 1,489,425 | 1,450,356 | 0·3358 | 0·2227 |
| 1944 | 45 | 47 | 1,503,333 | 1,554,071 | 0·2689 | 0·2727 |
| 1945 | 67 | 44 | 1,519,048 | 1,569,042 | 0·3917 | 0·2412 |
| 1946 | 66 | 58 | 1,537,951 | 1,586,298 | 0·3740 | 0·3225 |
| 1947 | 88 | 64 | 1,559,203 | 1,603,827 | 0·4780 | 0·3442 |
| 1948 | 106 | 45 | 1,577,632 | 1,621,368 | 0·5702 | 0·2390 |
| 1949 | 119 | 54 | 1,595,459 | 1,636,728 | 0·6320 | 0·2778 |
| 1950 | 150 | 66 | 1,618,324 | 1,645,664 | 0·7679 | 0·3387 |
| 1951 | 133 | 65 | 1,634,606 | 1,660,953 | 0·6860 | 0·3270 |
| 1952 | 161 | 82 | — | — | — | — |

FIG. 2.—Mortality from " lung cancer " per 10,000—Norway. Standard figures, 1929–51. Two curves have been drawn for females, the upper giving the figures standardized to the female population in 1930, the lower the figures standardized to the male population of 1930.

series (Christiansen (1953), 134 cases, and Jakobsen (1953), 100 cases), as well as the authors own clinical material, embracing 232 cases.

The examination of the histological types, as well as the sex and age occurrence, in the light of our present clinical and epidemiological knowledge lead to the following conclusions : (i) Squamous cell, large-cell and small-cell carcinomas represent a biological entity. These tumours occur with a very marked preponderance in males, and they show an age-curve indicating that in the so-called Western World potent carcinogenic factors of recent origin are of aetiologic significance. These tumours are designated Group I tumours. (ii) Adenocarcinomas occur in Norway with the same frequency in males and females, and this tumour shows a steadily increasing occurrence with advancing years, indicating a stable and moderate carcinogenic influence. (iii) Bronchiolar cell carcinomas are likewise evenly distributed among males and females, but there is no increase in the higher age-groups. Unknown factors hitting at random are supposed responsible for their development. (iv) Lung adenomas and salivary gland tumours of the lungs show an equal sex distribution and occur in all ages. These facts, together with the histological picture point in the direction of developmental factors being responsible for their occurrence. These last four tumour types have been designated Group II tumours.

If the three Norwegian materials are examined and Christiansen's (1953) series is divided into two sub-series, according to two different time periods, we get the figures as shown in Table II. A few cases, which occurred in both Christiansen and Kreyberg's materials, have been included in the latter only in this table.

TABLE II.

| | Group I tumours. | | Group II tumours. | |
|---|---|---|---|---|
| Material. | Males. | Females. | Males. | Females. |
| Christiansen (1953) covering the years 1925–1944 . . . . . . | 25 | 11 | 15 | 13 |
| Christiansen (1953) covering the years 1945–1952/1 . . . . . | 40 | 4 | 14 | 12 |
| Jakobsen (1953) covering the years 1937–1946 . . . . . . | 46 | 8 | 24 | 22 |
| Kreyberg (1954) covering the years 1948–1953 . . . . . | 162 | 8 | 28 | 29 |

Table II shows most strikingly the uniform occurrence of the Group II tumours in the two sexes in all the series, originating from different time periods. It should be emphasized that the present writer has been responsible for all the histological typing, and that the typing has been conducted at different periods and with no knowledge of the files, nor the clinical histories.

If the tentative conclusions presented above are correct, it would mean that the Group II tumours should represent primary lung tumours unrelated to special epidemiological circumstances, and that these tumours, with a possible reservation regarding the adenocarcinomas, should have the same relative frequency to-day as in the past. The adenocarcinomas may have increased in frequency in recent years, but the regular age-curve of this tumour type indicates that they have not very recently been influenced by external changes, in sharp contrast to the

Group I tumours. The consequence of this assumption should again be that an increase registered for Group II tumours would represent a measure of an increase in diagnostic efficacy.

As regards the Group I tumours, Table II again clearly reveals interesting facts. In the four time periods, the two middle partly overlapping, one may observe that females, in accord with clinical experience, constantly present a small number of Group I tumours. Compared now to the Group II tumours, they are at least not increasing in frequency. No conclusion in the opposite direction is drawn from the rather declining frequency because of the small figures.

It may therefore be correct to regard the lung cancer in females to-day as an expression of what Clemmesen, Nielsen and Jensen (1953) call " unavoidable " lung cancer. This unavoidable lung cancer should include all the Group II tumours, as well as a small number of Group I tumours.

If these assumptions are correct, the increase in lung cancer in females should at least in Norway today, be a fair index of the increased diagnostic efficacy. In the twenty years' period 1930–1950 this increase is approximately four-and-a-half-fold. We have, however, probably not yet reached the limitation of our diagnostic expansion. Although better diagnosis may in some cases lead to the discovery of a primary site outside the lung, and thereby exercise a depressive effect on the lung cancer mortality registered, it seems more likely that further improvements at the present time will mainly result in an increase in the mortality figures. We ought, therefore, to accept even a further increase in the lung cancer mortality registered in females, without necessarily drawing the conclusion that more lung cancer is actually developing. A very important clue as to a change in the situation is the ratio of the different histological types in the material.

If Table II and fig. 2 are correlated, one finds in males a different development taking place from the middle of the 1940's. From that period a marked increase in Group I tumours can be observed in males, at the same time as a pronounced deviation in the mortality rates in the two sexes takes place. It seems that the marked additional rise in lung tumours in males is caused solely by the Group I tumours. The Group II tumours, on the other hand, closely follow the pattern of the females. It may be that in the latest series (Kreyberg's) in Table II, the difference between the Group I and II tumours is too marked to be wholly representative, because that material is a clinical one with an underrepresentation of older people liable to develop adenocarcinomas. The tendency is clear, however, and the development continues to-day.

At present it seems permissible to conclude that in Norway, during the twenty years' period 1930–1950, the four-and-a-half-fold increase registered in lung cancer in females is entirely caused by greater diagnostic efficacy. As the development in males initially closely followed that of the females, it seems reasonable to accept the recent deviation in the mortality rates, as well as in the representation of the histological types, as indicating a new situation, and to regard the added increase in lung cancer in males as an expression of a real increase. This should mean the difference between a sevenfold and a four-and-a-half-fold increase.

The importance of this conclusion is not expressed by the magnitude of the real rise to-day, but it is indicated by the tendency and the short time it has been manifest. The increase in lung cancer in Norway has started later than in many other countries, but the pattern is identical.

SUMMARY.

The figures from the official Norwegian mortality statistics have been analysed on the background of the occurrence of different histological types of primary epithelial lung tumours in two Norwegian post-mortem materials and a series of clinical cases belonging to different time periods. It is found that in females the ratio of the different tumour types has not changed appreciably, and the increase in lung cancer in females is taken as indicator of the increased diagnostic efficacy. In males, on the other hand, a relative increase in the number of squamous cell, large-cell and small-cell carcinomas has taken place very recently, at the same time as a pronounced deviation in the mortality rates between the two sexes has developed. Accepting a four-and-a-half-fold increase in lung cancer in females as caused by better diagnosis, the difference between this figure and the sevenfold increase registered in males is taken to represent the real increase in the development of lung cancer in males.

The histological sub-division of the primary epithelial lung tumours into different histological and biological entities seems to be very useful in an analysis of the actual development of such tumours.

The present and the preceding paper were first read at the opening of our new Institute on March 16, 1953. Simultaneously, nearly identical observations and conclusions were in press in the important book ' Aetiologie und Prophylaxe des Lungenkrebses ' by F. Lickint (Dresden-Leipzig, 1953), unknown to the present author until our two papers were in press.

This study has been aided by a generous grant from " Tobaksfabrikernes Landsforening av 1901."

I am likewise greatly indepted to Dr. Knut Westlund for very valuable assistance during the preparation of the manuscript.

REFERENCES.

CHRISTIANSEN, T.—(1953) *Brit. J. Cancer*, **7**, 428.

CLEMMESEN, J., NIELSEN, A., AND JENSEN, E.—(1953) *Acta Un. int. Cancr.* **8**, Fasc. Spec., 160.

JAKOBSEN, A.—(1953) *Brit. J. Cancer*, **7**, 432.

KREYBERG, L.—(1952) *Ibid.*, **6**, 112—(1954) *Ibid.*, **8**, 199